# Artificial Intelligence in Dry Eye Disease: A Narrative Review

**DOI:** 10.7759/cureus.70056

**Published:** 2024-09-23

**Authors:** Praveena P Nair, Manjiri Keskar, Pramod T Borghare, Disha A Methwani, Yugandhara Nasre, Minakshi Chaudhary

**Affiliations:** 1 Ophthalmology, Mandsaur Institute of Ayurved Education and Research, Bhunyakhedi, IND; 2 Ophthalmology, Parul institute of Ayurved, Parul University, Limda, IND; 3 Otolaryngology, Mahatma Gandhi Ayurved College Hospital and Research, Wardha, IND; 4 Otolaryngology, NKP Salve Institute Of Medical Sciences & Research Centre And Lata Mangeshkar Hospital, Nagpur, IND; 5 Otolaryngology, BABM Nandanvan, Nagpur, Nagpur, IND; 6 Nursing, Shalinitai Meghe College of Nursing, Datta Meghe Institute of Higher Education and Research, Wardha, IND

**Keywords:** artificial intelligence, diagnosis, dry eye disease, machine learning, narrative review, ophthalmology, treatment

## Abstract

Dry eye disease (DED) is a multifactorial condition affecting millions worldwide, characterized by discomfort, visual disturbance, and potential damage to the ocular surface. The complexity of its diagnosis and management, driven by the diversity of symptoms and underlying causes, presents significant challenges to clinicians. Artificial intelligence (AI) has emerged as a transformative tool in healthcare, offering potential solutions to these challenges through its data analysis, pattern recognition, and predictive modeling capabilities. This narrative review explores the role of AI in diagnosing, treating, and managing dry eye disease. AI-driven tools such as machine learning algorithms, imaging technologies, and diagnostic platforms are examined for their ability to enhance diagnostic accuracy, personalize treatment approaches, and optimize patient outcomes. Furthermore, the review addresses the limitations of AI technologies in ophthalmology, including the need for robust clinical validation, data privacy concerns, and the ethical considerations of integrating AI into clinical practice. The findings suggest that while AI holds promise for improving the care of patients with DED, ongoing research and development are crucial to realizing its full potential.

## Introduction and background

Dry eye disease (DED) is a multifactorial disorder of the ocular surface characterized by a loss of homeostasis of the tear film, leading to symptoms of discomfort, visual disturbance, and tear film instability with potential damage to the ocular surface. It is a prevalent condition, affecting approximately 5-50% of the global population, with the variation in prevalence often attributed to differences in diagnostic criteria and demographic factors [1]. DED is associated with significant impacts on quality of life, including chronic pain, reduced work productivity, and limitations in daily activities such as reading and driving [2]. Additionally, patients with DED often suffer from emotional distress and an increased risk of developing mental health conditions, such as depression and anxiety [3].

The pathophysiology of DED is complex, involving various factors, including tear film instability, inflammation, neurosensory abnormalities, and ocular surface damage [4]. Two primary subtypes of DED have been identified: aqueous-deficient DED, where there is insufficient tear production, and evaporative DED, where tears evaporate too quickly [5]. Major risk factors for the development of DED include aging, female sex, hormonal changes, autoimmune diseases (e.g., Sjögren’s syndrome), environmental factors, and prolonged screen time [6]. Given its high prevalence and considerable burden, there is an ongoing need for improved diagnostic and treatment approaches to DED. Artificial intelligence (AI) refers to developing computer systems that can perform tasks typically requiring human intelligence, such as visual perception, speech recognition, decision-making, and data analysis [7]. In recent years, AI has revolutionized various aspects of healthcare, including disease diagnosis, treatment planning, and personalized medicine. AI technologies, particularly machine learning (ML) and deep learning (DL), have shown remarkable promise in analyzing complex datasets, identifying patterns, and providing actionable insights in medical practice [8].

Ophthalmology has been at the forefront of AI implementation in medicine. AI algorithms have been developed to aid in the diagnosis and management of various ocular diseases, such as diabetic retinopathy, glaucoma, and age-related macular degeneration (AMD) [9]. AI applications in ophthalmology often focus on image analysis from diagnostic tools like optical coherence tomography (OCT), fundus photography, and corneal topography [10]. This growing body of work has demonstrated that AI can improve diagnostic accuracy, reduce time to diagnosis, and facilitate earlier interventions [11]. Given the increasing burden of DED and the limitations of current diagnostic methods, the application of AI in diagnosing and managing DED holds significant potential. AI-driven models can assist in analyzing large volumes of data, such as tear film dynamics, patient-reported symptoms, and imaging results from ocular surface analysis. Furthermore, AI tools could enhance clinical decision-making by predicting disease progression and optimizing individualized treatment plans [12]. The rationale for applying AI in DED is grounded in its ability to improve the precision and efficiency of care, ultimately leading to better patient outcomes.

## Review

Search methodology

The search methodology for this narrative review on "Artificial Intelligence in Dry Eye Disease" involved a comprehensive literature search across multiple databases, including PubMed, Scopus, and Google Scholar. The search terms used were a combination of keywords such as "artificial intelligence," "machine learning," "deep learning," "dry eye disease," "ophthalmology," "diagnosis," and "treatment." Articles published in English from 2010 to 2024 were included to capture the most recent advancements. Studies focusing on the application of AI in the diagnosis, management, and treatment of dry eye disease were considered with priority given to peer-reviewed original research, reviews, and clinical trials. Exclusion criteria included studies unrelated to AI or dry eye disease, animal studies, and non-English publications. Additionally, reference lists of key articles were manually searched to identify further relevant studies.

Current diagnosis and management of dry eye disease

Traditional Diagnostic Methods

Clinical examinations: Diagnosing dry eye disease (DED) traditionally relies on clinical examinations, with several well-established tests commonly used. One such test is the Schirmer test, which measures tear production by placing a strip of filter paper under the lower eyelid. A tear film below 5 mm after five minutes generally indicates dry eye. Another common clinical measure is tear film breakup time (TBUT), where a fluorescein dye is applied to the tear film to assess how quickly the tear film breaks up after a blink. A breakup time shorter than 10 seconds suggests tear film instability, a hallmark of DED [13].

Imaging and biomarkers: In addition to clinical tests, advancements in imaging techniques have enhanced diagnostic accuracy. Ocular surface stainings, such as fluorescein and lissamine green, allow for visualizing damaged epithelial cells. More advanced tools, such as meibography, provide detailed images of the meibomian glands to assess gland dysfunction, a major contributor to evaporative dry eye [14]. Biomarkers like MMP-9, a matrix metalloproteinase, are also increasingly used to diagnose the tear film's inflammatory changes [15].

Challenges in Diagnosing and Managing DED

Subjectivity in diagnosis: Despite the availability of these diagnostic methods, the diagnosis of DED is still highly subjective. Patient-reported symptoms, which can be inconsistent and vary significantly among individuals, often serve as a major component of the diagnosis. Objective clinical signs of dry eye usually do not correlate well with the severity of symptoms, making diagnosis difficult [16].

Limited precision in current methods: Current diagnostic tools also suffer from limited precision. For example, the Schirmer test and TBUT provide only a general assessment and are affected by external factors such as room humidity and patient anxiety. This lack of precision hinders accurate disease severity staging and treatment efficacy tracking [17].

Need for personalized treatment strategies: The complexity of DED, with its multifactorial causes and varied patient presentations, makes it challenging to manage effectively using a one-size-fits-all approach. Treatment typically focuses on alleviating symptoms rather than addressing the underlying pathology. A more personalized approach, taking into account individual tear film composition, inflammation levels, and meibomian gland health, is urgently needed [18].

Application of AI in dry eye disease

AI-Based Diagnostic Tools

AI-driven imaging techniques: Meibography, which provides images of the meibomian glands in the eyelids, is crucial for diagnosing meibomian gland dysfunction (MGD), a common cause of DED. AI-powered tools have been developed to interpret these images, identifying gland atrophy and abnormalities accurately. For instance, machine learning algorithms can detect gland dropout and other pathologies that may go unnoticed during manual assessment [19]. Similarly, AI has been integrated into tear film analysis, which assesses the stability and quality of the tear film, a critical component in diagnosing DED. Techniques such as non-invasive tear break-up time (NITBUT) can now be analyzed through AI-driven systems, which process high-resolution images to detect minute changes in the tear film, offering real-time, accurate diagnostics [20].

Machine learning models for predictive diagnosis: Machine learning (ML) models are proving invaluable in predictive diagnostics for DED. These models can analyze large datasets, including patient demographics, clinical symptoms, and lifestyle factors, to predict the likelihood of developing DED or the potential severity of the condition. For instance, ML algorithms have been trained to recognize patterns in ocular surface metrics and tear film characteristics to forecast DED progression. This enables earlier interventions and tailored management plans for at-risk patients [21].

AI in Personalized Treatment Plans

AI-powered algorithms for individualized therapy: AI-powered algorithms can analyze patient-specific data, such as ocular surface characteristics, tear production levels, and meibomian gland function, to generate personalized treatment recommendations. These algorithms consider various treatment modalities, such as artificial tears, anti-inflammatory medications, and thermal treatments, and suggest the most effective combinations based on the individual’s unique presentation of DED. In recent studies, AI tools have been employed to recommend tailored therapies, improving the precision and efficacy of treatment while reducing trial and error in management [22].

Monitoring and follow-up care using AI technologies: AI also plays a role in continuously monitoring DED patients. AI-driven apps and wearable devices are now being used to track symptoms, such as discomfort and tear film stability, between clinical visits. These technologies allow for remote monitoring and real-time feedback, enabling timely adjustments in treatment plans. Furthermore, AI-based platforms can predict treatment outcomes and flag potential complications early, improving patient adherence and long-term disease management [23].

AI Techniques in DED diagnosis and treatment

*Machine Learning-* *Overview of Supervised, Unsupervised, and Deep Learning Approaches*

Supervised learning: It involves training algorithms on labeled data, where the outcome is already known, to predict future instances. Supervised learning is widely used in DED research to classify disease severity based on clinical parameters and images [24].

Unsupervised learning: This technique works with unlabeled data, finding hidden patterns or groupings. It can be applied in DED to detect disease subtypes or identify patient clusters based on tear film characteristics [25].

Deep learning: A subset of machine learning, deep learning mimics human neural networks and excels in handling large volumes of complex data. Deep learning models have demonstrated considerable accuracy in diagnosing ocular surface conditions and are promising in DED applications [20].

Application of Machine Learning Models in Tear Film Analysis and Meibomian Gland Imaging

ML models have been applied to analyze tear film metrics and meibomian gland images, which are crucial in diagnosing DED. For example, support vector machines (SVM) and random forest algorithms have been employed to analyze tear breakup time, osmolarity, and lipid layer thickness [26]. In meibomian gland imaging, ML algorithms like k-means clustering and deep neural networks have been used to detect and assess gland morphology, which helps in diagnosing and classifying DED subtypes [27].

Deep Learning and Convolutional Neural Networks (CNNs)

Use of CNNs in interpreting ocular surface images: Convolutional neural networks (CNNs) are a robust deep learning architecture that is well-suited for image-based tasks. CNNs have been extensively applied to ocular surface imaging in DED. These networks can automatically extract features from raw images, such as tear meniscus height and corneal staining patterns, leading to accurate diagnosis without manual intervention [28]. One notable example is their use in analyzing meibography, where CNNs have shown high accuracy in detecting meibomian gland dysfunction (MGD) in DED patients [29].

Role of deep learning in classifying DED severity: Deep learning models have also been instrumental in categorizing the severity of DED. Researchers have trained CNNs on clinical images and objective measurements (e.g., Schirmer’s test, tear breakup time) to categorize DED into mild, moderate, and severe cases. These models achieve a high degree of accuracy in classification, reducing reliance on subjective clinical assessments and enabling more standardized and precise diagnoses [30].

AI in Predicting Disease Progression

Predictive modelling using large datasets of DED patients: Predictive modeling is another critical area where AI has shown great promise in DED. By analyzing large datasets from diverse patient populations, machine learning algorithms can predict the progression of DED and assess patient outcomes. Models using gradient boosting machines (GBM) and artificial neural networks (ANN) have been trained on various clinical parameters, such as tear volume, osmolarity, and ocular surface inflammation markers, to forecast disease exacerbation or remission over time [21]. These predictive tools are invaluable for personalized treatment planning, allowing clinicians to adjust therapies based on an individual's predicted trajectory.

Current research and advances in AI for DED

Ongoing Studies and Clinical Trials

Recent advancements in artificial intelligence (AI) have significantly influenced the field of dry eye disease (DED). Numerous studies and clinical trials have emerged, focusing on leveraging AI for improved diagnosis and personalized treatment of DED. It explored using deep learning algorithms to analyze tear break-up time (TBUT) and corneal staining images. The study demonstrated that AI could enhance the accuracy of DED diagnosis by 15% compared to traditional methods, highlighting the potential of AI to provide more precise assessments in clinical settings [31]. Another significant study investigated AI-powered algorithms for predicting the response of DED patients to different treatments. By analyzing patient data, including symptom questionnaires and clinical measurements, the AI model could forecast treatment outcomes with a high degree of accuracy. This predictive capability could lead to more personalized and effective treatment strategies [21].

Innovations in AI-Driven Diagnostic Devices

Smart devices for home-based tear film monitoring: Recent innovations have led to the development of smart devices designed for monitoring. One notable example is the TearScan® device, which uses AI algorithms to analyze tear film stability and quality from home settings. The device provides patients with real-time feedback and recommendations, enabling better condition management without frequent clinic visits [25].

Mobile apps and telemedicine supported by AI for DED management: The rise of mobile apps and telemedicine platforms supported by AI has transformed DED management. AI-powered mobile apps, such as DryEyeDoc™ and EyeCheck™, offer tools for symptom tracking, image analysis, and treatment recommendations. These apps use machine learning algorithms to analyze user-reported symptoms and imaging data, providing personalized management strategies and facilitating remote consultations with healthcare professionals [32].

Limitations and challenges of AI in dry eye disease

Data Limitations

Lack of large, diverse datasets for AI training: AI models, particularly those utilizing deep learning techniques, require large and diverse datasets to be trained effectively. In the context of dry eye disease (DED), the availability of such datasets is limited. Studies often rely on small, homogeneous patient populations, which can lead to overfitting and limit the generalizability of AI algorithms [33]. Moreover, the heterogeneity in DED symptoms and patient responses necessitates a broader dataset to ensure that AI models can effectively account for variability in clinical presentations [12].

Issues of data quality and variability in DED patients: The quality and consistency of data used to train AI systems can significantly impact their performance. In DED research, variability in diagnostic criteria, subjective symptom assessments, and treatment outcomes can introduce noise into the data [34]. This variability can undermine the reliability of AI tools, making it challenging to develop robust models across different patient populations and clinical settings [35]. Furthermore, the lack of standardized protocols for data collection in DED contributes to discrepancies and limits the effectiveness of AI systems.

Technological Barriers

Integration of AI tools in clinical practice: Integrating AI tools into existing clinical workflows presents a substantial challenge. Many healthcare settings lack the necessary infrastructure to support advanced AI technologies [36]. Additionally, there is a need for seamless integration with electronic health records (EHRs) and other clinical systems to ensure that AI tools can be used effectively in day-to-day practice [37]. Without such integration, the adoption of AI in clinical settings may be limited, affecting its potential to improve patient care.

High costs and the need for specialized training: The development and implementation of AI tools in DED are associated with high costs, including expenses for software development, hardware requirements, and ongoing maintenance. Furthermore, healthcare professionals need specialized training to effectively utilize these AI tools, which can be a barrier to widespread adoption. The financial and educational investments required may limit the accessibility and practical use of AI technologies in many healthcare environments [38].

Ethical and Legal Concerns

Privacy and security of patient data: The use of AI in healthcare raises significant concerns about the privacy and security of patient data. AI systems require access to sensitive health information, which must be protected to prevent breaches and unauthorized access. Ensuring robust data protection measures and complying with regulations such as the General Data Protection Regulation (GDPR) are critical to safeguarding patient information [39]. However, implementing these measures can be complex and resource-intensive.

Ethical implications of AI decision-making in DED care: AI decision-making in DED care brings up ethical questions related to accountability and transparency. There is a concern about how decisions made by AI systems are communicated to patients and how these decisions are justified. Additionally, the potential for AI to exacerbate existing disparities in healthcare access and outcomes must be addressed [40]. Ethical frameworks and guidelines are needed to ensure that AI technologies are used responsibly and equitably in treating dry eye disease.

Future directions and potential of AI in dry eye disease

AI for Early Detection and Prevention

Potential for AI to detect early stages of DED before symptoms manifest: The advent of AI offers promising potential for the early detection of dry eye disease (DED) before the onset of overt symptoms. Recent advancements in AI and machine learning algorithms have demonstrated the capacity to analyze vast datasets from patient records and diagnostic tools to identify subtle patterns indicative of early-stage DED. For instance, AI algorithms have been employed to assess ocular surface images and identify microscopic changes that precede symptomatic DED [21]. By leveraging these capabilities, clinicians can initiate preventive measures earlier, potentially mitigating disease progression.

Predictive analytics for preventive care: Predictive analytics, powered by AI, can enhance preventive care strategies for DED. AI models, trained on comprehensive patient data, including demographic, environmental, and clinical variables, can forecast individual risk profiles for developing DED. Such predictive tools can inform tailored preventive interventions, such as lifestyle modifications and targeted therapies [20]. The application of AI in predictive analytics not only aids in early intervention but also enhances the overall management of DED by reducing the incidence and severity of the disease.

AI in Patient-Centered Care

Enhancing patient engagement through AI-driven tools: AI-driven tools have the potential to significantly enhance patient engagement in managing DED. AI-powered applications and platforms can provide personalized education and real-time patient feedback, fostering a more proactive approach to self-care. For example, AI-based mobile applications can offer individualized recommendations for lifestyle changes, medication adherence, and symptom monitoring, improving patient involvement and adherence to treatment regimens [41].

Role of AI in remote monitoring and telemedicine for DED: Integrating AI in telemedicine offers transformative possibilities for managing DED. AI-powered remote monitoring systems can track symptoms and treatment responses through wearable devices and telehealth platforms, providing continuous care without frequent in-person visits. Such systems facilitate real-time data collection and analysis, enabling timely adjustments to treatment plans and improving overall patient outcomes [42]. The expansion of AI in telemedicine can bridge gaps in access to care, especially for patients in underserved areas.

Integration of AI with Other Emerging Technologies

Combining AI with wearable sensors, virtual reality (VR), and robotics for advanced DED care: The convergence of AI with emerging technologies holds great promise for advancing DED care. Wearable sensors with AI algorithms can continuously monitor ocular parameters and environmental factors, providing dynamic insights into disease progression and treatment efficacy [43]. Additionally, virtual reality (VR) can be used to simulate various ocular conditions and treatment responses, enhancing patient education and engagement. In combination with AI, robots can offer precision in therapeutic interventions, such as targeted drug delivery systems or advanced surgical techniques [44]. Integrating these technologies with AI will likely drive significant improvements in the diagnosis, treatment, and management of DED.

## Conclusions

In conclusion, artificial intelligence (AI) has shown promising potential in the diagnosis, management, and treatment of dry eye disease (DED). AI-driven technologies, including machine learning algorithms and advanced imaging tools, offer more precise and efficient diagnostic capabilities, enabling early detection and personalized treatment plans. These innovations have the potential to enhance clinical decision-making, reduce the burden on healthcare providers, and improve patient outcomes. However, challenges such as data privacy, the need for large-scale validation studies, and integration into clinical workflows must be addressed to fully realize AI's benefits in the field of DED.
